# Identification by Exome Sequencing of Predisposing Variants in Familial Cases of Autoinflammatory Recurrent Fevers

**DOI:** 10.3390/genes14071310

**Published:** 2023-06-21

**Authors:** Eugenio Sangiorgi, Alessia Azzarà, Roberto Rumore, Ilaria Cassano, Elena Verrecchia, Luciano Giacò, Maria Alessandra Tullio, Fiorella Gurrieri, Raffaele Manna

**Affiliations:** 1 Dipartimento di Scienze della Vita e Sanità Pubblica, Sezione di Medicina Genomica, Università Cattolica del Sacro Cuore–Fondazione Policlinico Universitario A. Gemelli IRCCS, 00168 Roma, Italy; roberto_rumore@hotmail.it (R.R.); ilariacassano@outlook.it (I.C.); 2 Research Unit of Medical Genetics, Department of Medicine and Surgery, Università Campus Bio-Medico di Roma, 00128 Rome, Italy; a.azzara@unicampus.it (A.A.); f.gurrieri@unicampus.it (F.G.); 3 Dipartimento di Scienze Geriatriche e Ortopediche, Università Cattolica del Sacro Cuore–Fondazione Policlinico Universitario A. Gemelli IRCCS, 00168 Roma, Italy; elena.verrecchia@policlinicogemelli.it; 4 Bioinformatics Core Facility, Gemelli Science and Technology Park (G-STeP), Fondazione Policlinico Universitario A. Gemelli IRCCS, 00168 Roma, Italy; luciano.giaco@policlinicogemelli.it (L.G.); ma.tullio@gmail.com (M.A.T.); 5 Operative Research Unit of Medical Genetics, Fondazione Policlinico Universitario Campus Bio-Medico, 00128 Rome, Italy; 6 Periodic Fevers Research Center, Università Cattolica del Sacro Cuore–Fondazione Policlinico Universitario A. Gemelli IRCCS, 00168 Roma, Italy; raffaele.manna@policlinicogemelli.it

**Keywords:** autoinflammatory diseases, exome sequencing, periodic fever

## Abstract

Periodic fever syndromes include autoinflammatory disorders (AID) that involve innate immunity. These disorders are characterized by recurrent fevers and aberrant multi-organ inflammation, without any involvement of T or B cells or the presence of autoantibodies. A complex genetic architecture has been recognized for many AID. However, this complexity has only been partially uncovered for familial Mediterranean fever and other conditions that have a classical monogenic origin and Mendelian transmission. Several gene panels are currently available for molecular diagnosis in patients suspected of having AID. However, even when an extensive number of genes (up to 50–100) are tested in a cohort of clinically selected patients, the diagnostic yield of AID ranges between 15% and 25%, depending on the clinical criteria used for patient selection. In the remaining 75–85% of cases, it is conceivable that the causative gene or genes responsible for a specific condition are still elusive. In these cases, the disease could be explained by variants, either recessive or dominant, that have a major effect on unknown genes, or by the cumulative impact of different variants in more than one gene, each with minor additive effects. In this study, we focused our attention on five familial cases of AID presenting with classical autosomal dominant transmission. To identify the probable monogenic cause, we performed exome sequencing. Through prioritization, filtering, and segregation analysis, we identified a few variants for each family. Subsequent bioinformatics evaluation and pathway analysis helped to narrow down the best candidate genes for each family to *FCRL6*, *PKN1*, *STAB1*, *PTDGR*, and *VCAM1*. Future studies on larger cohorts of familial cases will help confirm the pathogenic role of these genes in the pathogenesis of these complex disorders.

## 1. Introduction

Autoinflammatory conditions (AID) are a group of diseases with a strong genetic component [1,2]. The identification of the MEFV gene in familial Mediterranean fever families in 1997 [3,4] led to discoveries of many other genes in different AID conditions. Along with monogenic entities, there are other mixed autoinflammatory disorders, such as Behçet’s disease, Kawasaki syndrome, and recurrent pericarditis, with a more complex genetic architecture requiring the intervention of multiple variants in more than one gene, along with other endogenous and exogenous factors [5,6]. For this reason, they are rarely transmitted in a Mendelian way. When AIDs present in a sporadic patient, it can be difficult to determine whether they have monogenic or more complex origins. Many autoinflammatory conditions are often diagnosed based solely on the presence of recurrent fever and, after ruling out variants in major genes through the analysis of a gene panel, it can be challenging to molecularly define a specific diagnosis [7]. These cases are now labeled as syndromes of undefined recurrent fever (SURF), where a polygenic or complex genetic-environmental architecture can be suspected [8,9,10]. A subgroup of SURF patients presents a well-defined autosomal dominant Mendelian transmission, highlighting a major gene variant in those cases.

The diagnosis of SURF overlaps with the clinical diagnosis of PFAPA. This syndrome, first described in 1987 [11], is characterized by febrile attacks that occur with “clockwork” periodicity and are accompanied by stereotyped symptoms in the oral cavity, such as tonsillitis/oral aphthosis and/or lymph node enlargement. It is predominantly observed in children, and approximately 10% of cases have an autosomal dominant transmission, highlighting its strong genetic origin. Consequently, most genetic studies have focused on this condition. In a comprehensive genetic study involving 14 families, a locus on chromosome 8 was identified through linkage analysis [12]. However, subsequent whole-exome sequencing failed to reveal any significant variants in the same locus or elsewhere in the exome. The same research group conducted targeted sequencing of 32 genes involved in innate immunity in 82 patients diagnosed with PFAPA [13]. This analysis revealed a frameshift variant in CARD8, which displayed a significant association with the phenotype. Additionally, other susceptibility loci were identified by searching for common predisposing variants of other oropharyngeal ulcerative disorders [14]. Our research group identified rare variants in the ALPK1 gene in a few individuals exhibiting PFAPA-like symptoms [15]. Nevertheless, the majority of patients diagnosed with PFAPA or SURF still lack a genetic diagnosis. For this reason, we focused our attention on those cases that presented a documented familial history of recurrent fever along with other symptoms. In order to investigate the responsible gene in SURF/PFAPA families, we undertook whole-exome sequencing analysis in two affected individuals from each of the five families we identified. This analysis, followed by the bioinformatics characterization of the variants and genes, allowed us to identify different putative predisposing variants in different genes involved in autoinflammation, highlighting the broad genetic heterogeneity of these conditions.

## 2. Material and Methods

### 2.1. Families

All patients and families were recruited at our outpatient clinics, “Centro per lo studio delle Febbri Periodiche,” at the Fondazione Policlinico Gemelli. For each proband, their clinical history was evaluated, and biochemical and blood values were recorded during each visit. Biochemical tests and blood analyses were conducted during and after fever recurrences as part of the clinical management.

**Figure 1:**
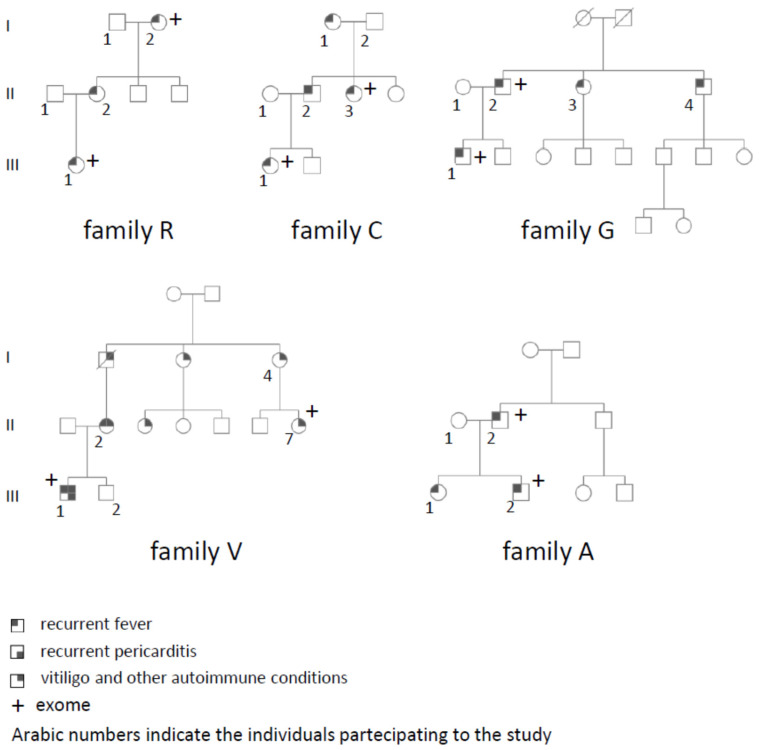
Pedigrees of the families participating in this study. In each family, individuals with an Arabic numeral participated in this study, providing their DNA upon signing the informed consent form. Individuals with «+» had their exome sequenced.

During the enrollment process, a comprehensive familial history was obtained. When additional individuals with similar symptoms were reported, we contacted and recruited them. We extended the genetic analysis to all available family members whenever possible. Five families were recruited (Figure 1). All individuals provided informed consent, which was approved in 2020 by the Comitato di Bioetica of Fondazione Policlinico Gemelli. All five families had at least two or more affected individuals and tested negative for variants in a gene panel of 13 genes involved in autoinflammatory diseases. Families R, C, G, and A had a clinical diagnosis of PFAPA, SURF, or AIDs not otherwise specified. Family V had a much more complex phenotype with autoinflammatory and autoimmune symptoms. Individual III1 had recurrent fever along with Henoch–Schoenlein purpura and two episodes of pericarditis. His main problem was represented by demyelinating lesions in his brain. His mother, II1, had a series of autoimmune conditions (vitiligo, Hashimoto’s thyroiditis, autoimmune gastritis, and recurrent sterile tonsillitis), her father, I1, died as a consequence of a polyneuritis and transverse myelitis, while the remaining individuals were reported to have only vitiligo.

### 2.2. Sequencing

Whole-exome sequencing was performed by the Galseq SRL company (Bresso, Milan, Italy) using Illumina technology. An average coverage of 60X was obtained, and paired fastq files were processed on the Galaxy server following standard procedures [16]. After variant calling, we isolated high-quality variants shared by the two affected individuals in each family and annotated them. We selected variants in exonic/splicing regions and filtered out synonymous variants, common variants (MAF > 1/1000), and homozygous variants, considering the apparent autosomal dominant transmission. From the Infevers database (https://infevers.umai-montpellier.fr/ accessed on 20 June 2023) the list of genes involved in autoinflammatory conditions was downloaded. Variants were then prioritized based on keywords such as “autoinflammatory condition,” “inflammasome,” “innate immunity,” “IL1,” “MEFV,” “PYRIN,” and “autoinflammation” using the Varelect software (https://varelect.genecards.org/ accessed on 20 June 2023) [17]. Variants in the top 20 genes according to Varelect were then segregated in the remaining affected individuals of the family, and those not segregating were no longer considered for bioinformatics evaluation.

Sanger sequencing was performed on each variant that passed the filtering/prioritization procedure using standard methods. The variants were analyzed in the affected individuals of the same family.

### 2.3. Bioinformatics Analysis

A bioinformatics analysis of the genes was conducted using the COXPRESdb (https://coxpresdb.jp/ accessed on 20 June 2023) database [18], which investigates genes co-expressed across different cell types, tissues, and experimental settings to identify genes co-expressed in different gene networks. Gene co-expression represents the synchronization of gene expression across various cellular and environmental conditions and is widely used to infer the biological function of genes. We uploaded our genes along with genes from the Infevers database to identify which genes from our families were co-expressed with genes already identified in other autoinflammatory conditions.

The STRING database (https://string-db.org/ accessed on 20 June 2023) [19], one of the main resources dedicated to organism-wide protein association networks, was used to create a protein–protein interaction network based on the genes detected in the five families, which could be the hypothetical cause of recurrent chronic fevers with inheritance characteristics, and the genes derived from the Infevers database. The minimum required interaction score was lowered to 0.40 (medium confidence) to allow the connection between all the inserted genes/nodes in the multiple proteins selection.

## 3. Results

We recruited five families with a clinical history characterized by recurrent fever attacks in two or more individuals (Figure 1). Based on pedigree analysis, putative autosomal dominant transmission was suspected. For each family, we selected the two most distantly related individuals for exome sequencing. To prioritize variants in genes already involved in autoinflammatory conditions, we used a gene list for autoinflammatory diseases derived from the Infevers database. However, no pathogenic variants were identified in any of those genes. We then used the Varelect analysis to identify genes prioritized based on keywords related to autoinflammatory conditions. The variants in the top 20 genes with the highest score from this analysis were confirmed using Sanger sequencing and were segregated among all the other affected individuals in the family. Variants shared by all the affected individuals had their frequency evaluated in the gnomAD database (https://gnomad.broadinstitute.org/ accessed on 20 June 2023) and their in silico impact was analyzed using the CADD score [20] and other common software (Table 1).

In family R, four variants in four different genes were identified to segregate with the phenotype (Table 1). As one of the genes was the C-reactive protein (CRP) gene, we investigated whether rare variants in this gene were responsible for a similar phenotype in other sporadic or familial cases of autoinflammatory diseases (AID) in our database. This family had fever periodicity characterized by episodes lasting 7–10 days every 2 months, similar to TNF receptor-associated periodic syndrome (TRAPS) [21,22]. We screened 150 patients, 40 of whom had suspected TRAPS, but none of them carried any rare variant in the CRP gene. In family G, four candidate variants were segregating, while in families C, V, and A, three different variants in each family segregated with the phenotype. Families A and V shared two variants in the same gene PNN (Table 1). To sort the strongest candidate among the new genes identified in these five families, we used bioinformatics tools to determine the role and expression of those genes with respect to genes already involved in autoinflammatory diseases. Firstly, we determined the expression domain in cells and organs involved in the pathogenesis of the disease using the online server COXPRESdb. Three genes, *FCRL6*, *TCTEX1D4*, and *PBK*, were not found to be highly expressed in the hematopoietic system. From this analysis, eight genes (*GBP3*, *CCNG2*, *STAB1*, *PTGDR*, *PNN*, *VCAM1*, *PKN1*, *GNAI2*) showed a high level of expression in whole blood, spleen, tonsils, lymph nodes, or bone marrow (Table 2). Using the same database, we investigated whether the proteins obtained from our exome analysis were interacting with the proteins in the Infevers database. From this analysis, GNAI2, PKN1, and VCAM1 were found to directly interact with proteins in the Infevers datasets (Table 3).

To further extend our evaluation of the protein–protein interaction network, we interrogated the STRING database. We uploaded the genes obtained from our exome analysis in node 1 and the genes from the Infevers database in node 2. Using a threshold for the combined score of above 0.4, the five genes with the strongest interactions with one or multiple proteins were *GNAI2*, *PKN1*, *CRP*, *SLC15A1*, and *GBP3* (Table 4).

## 4. Discussion

In this study, we performed exome sequencing on five families diagnosed with SURF [8,9,10], PFAPA [23,24,25], or atypical PFAPA syndrome. Due to the limited size of each family, we were unable to restrict the number of segregating variants to a single one through segregation analysis among the affected individuals. However, we utilized bioinformatics tools, expression data, and a literature search to infer stronger candidates for each gene. We prioritized variants with a lower allelic frequency and a higher CADD score, and then evaluated their expression level, involvement in the innate immune system, and strong interaction in the autoinflammatory pathway through co-expression and direct protein-protein interactions.

In family R, the *CRP* gene, initially considered a potential candidate, was partly excluded as we did not find any additional variants in a large cohort of familial and sporadic AID patients. Moreover, the identified variant had a relatively high frequency in the gnomAD database. Consequently, we directed our focus towards three remaining variants in the *MROH9*, *FCRL6*, and *KIF26B* genes. Among these, *FCRL6* emerged as the most promising candidate due to its strong expression and role in the immune system [26]. The encoded protein is an Fc-receptor-like protein highly expressed on cytotoxic T and NK cells [27]. It is upregulated on the expanded population of terminally differentiated CD8^+^ T cells in patients with HIV and B-cell chronic lymphocytic leukemia. However, its role in the autoinflammatory process remains to be determined, as no direct or indirect interactions with proteins/pathways involved in autoinflammation were retrieved from the bioinformatics analyses using the Infevers genes.

In family G, we identified four variants in four different genes, with the strongest candidate being the *PKN1* gene. The p.(Gly884Ser) variant in *PKN1* is novel with a high CADD score, placing it in the top 1% of deleterious variants. This variant is located in the AGC-kinase C-terminal domain of the protein [28]. PKN1 is a kinase that phosphorylates PYRIN at serine positions 242 and 244, blocking its activation [29,30]. Consequently, PKN1 emerged as the strongest candidate based on bioinformatics evaluations from different databases. Its role should be further evaluated in other familial and sporadic patients with autoinflammatory conditions to validate its significance.

In family C, three variants were identified, among which the variants in *STAB1* and *CCNG2* looked promising. On the other hand, the *GNAI2* variant had a low CADD score, indicating a more benign role. Both variants in *STAB1* and *CCNG2* had CADD scores above 20, placing them in the top 1% of deleterious variants. *CCNG2* is a cyclin with a role in cell-cycle regulation in normal and cancer cells, but no specific role has been described in regulating inflammation or the immune system thus far [31,32]. *STAB1* is a scavenger receptor expressed in many immune cells, especially macrophages, with a central role in the innate immune system [33]. Knockout (KO) mice for *Stab1* are more susceptible to the lethal effects of Coxsackievirus B3-induced myocarditis, sepsis, or Listeria infection [34,35]. Additionally, KO mice spontaneously develop an autoimmune phenotype as they age. The presumed mechanism is through blocking the fibronectin-mediated STAB1^+^ monocyte recruitment and differentiation into anti-inflammatory macrophages, resulting in an increased T cell response. Loss-of-function mutations could lead to a dysregulated inflammatory response both in animal models and in humans.

In family V, we observed two interesting variants among three genes, both with CADD scores close to 30 and functions in the innate immune system. The first gene, PNN, was the only gene found to be mutated in two different families in our cohort of five families. This gene, called PININ, is involved in desmosome junctions and splicing mechanisms [36]. In mice, its mutation leads to early embryonic lethality in the homozygous state, while a hypomorphic allele causes widespread defects in neural crest derivatives and consequent malformations in different organs and tissues [37]. Therefore, it is an unlikely candidate for autoinflammatory diseases. The stronger candidate in this family is the *PTGDR* gene, encoding a receptor for Prostaglandin D2. Polymorphisms in this gene have been controversially associated with predisposition to asthma and allergic reactions. The p.(Met228Ile) variant is very rare in the gnomAD database and has a CADD score of 29. The PGA2/PTGDR axis is considered to have an anti-inflammatory role. PTGDR has an inhibitory role in PYDC3, a protein that negatively regulates inflammasome activation [38]. Ptgdr^−/−^ mice exhibit increased IL-1β expression with increased mortality when challenged with a neurotropic virus. This effect was mediated by increased activation of the inflammasome and reduced by blocking IL1β [39]. It is interesting to observe that this protein is considered critical for the pathogenesis of experimental autoimmune encephalitis, an animal model for multiple sclerosis. In family V, individuals III1 and I2, along with other symptoms have signs and symptoms of a demyelinating disease.

In family A, after excluding *PNN* due to its role in development, we focused our attention on the *VCAM1* variant. This variant has a high CADD score and has never been reported before in the gnomAD database. It falls within the first Ig-like domain of the protein, adjacent to a cysteine required to form disulfide bonds. VCAM1 is an adhesion molecule initially identified as an endothelial cell surface glycoprotein [40]. It binds α4β1 and α4β7 integrins on leukocytes through Ig-like domains 1 and 4, facilitating rolling, adhesion, and transmigration. VCAM1 expression is driven by TNFα through the NFKB and MAPK pathway, and its expression facilitates leukocyte migration through endothelial junctions. This protein is at a crossroads with most of the pathways involved in autoinflammatory conditions and in the innate immune system [40].

In summary, through exome sequencing, we identified potential candidate genes for each family, selected based on the variant and their involvement in the innate immunity. These five genes, namely *FCRL6*, *PKN1*, *STAB1*, *PTGDR*, and *VCAM1*, hold significant promise as candidates for further investigation in future studies involving a larger cohort of patients with autoinflammatory diseases.

## Figures and Tables

**Table 1:** List of the variants identified in different family segregating among the affected individuals. The allelic frequency of each variant was obtained from the gnomAD database. In silico evaluations of each variant were performed using CADD, Mutation Taster, SIFT, Polyphen and GERP.

Genes	Variant	Allelic Frequency gnomAD	CADD	Mutation Taster	SIFT	Polyphen	GERP	
*MROH9*	p.Leu671Ser	6/179018	23.6	Polymorphism	Damaging	Probably damaging	5.91	family R
*CRP*	p.Arg206Trp	77/282680	19.27	Polymorphism	Damaging	Possibly damaging	−2.18	
*FCRL6*	p.Gly350Arg	23/282834	14.48	Polymorphism	Tolerated	Possibly damaging	−1.12	
*KIF26B*	p.Ala277Val	47/269910	14.42	Polymorphism	Tolerated	Benign	3.9	
*NUBP1*	p.Pro5Arg	46/208086	32	Disease causing	Deleterious	Probably damaging	3.96	family G
*SLC15A1*	p.Ile631Thr	313/282220	25.9	Disease causing	Deleterious	Benign	5.91	
*PKN1*	p.Gly884Ser	-	23.6	Disease causing	Tolerated	Benign	3.82	
*GBP3*	p.Glu457Asp	223/282744	14.52	Polymorphism	Damaging	Benign	0.45	
*CCNG2*	p.Ala67Val	-	23.7	Polymorphism	Damaging	Benign	4.55	family C
*STAB1*	p.Arg1305Gln	19/250642	21.6	Polymorphism	Tolerated	Benign	0.26	
*GNAI2*	p.Thr11Lys	-	6.59	Disease causing	Damaging	Benign	−0.36	
*PTGDR*	p.Met228Ile	2/245746	29.3	Disease causing	Damaging	Probably damaging	4.14	family V
*PNN*	p.Ala74Pfs*43	-	28.6	Disease causing	-	-	-	
*TCTEX1D4*	p.Gly115Glu	-	11.28	Polymorphism	Tolerated	Benign	3.5	
*PNN*	p.Asp680Gly	1/251262	28.6	Disease causing	Damaging	Probably damaging	6.13	family A
*VCAM1*	p.Thr49Ile	-	23.6	Polymorphism	Damaging	Possibly damaging	4.73	
*PBK*	p.Asp178Asn	7/279908	23	Disease causing	Tolerated	Possibly damaging	2.24	

**Table 2:** Gene expression values obtained from the COXPRESdb, using the GSE3526 and GTEx data. The replicates for each of the tissues were averaged in base-2 logarithm. Different gray shades indicates higher expression values.

	GSE3526				GTEx	
Gene	Bone Marrow	Lymph Nodes	Spleen	Tonsil	Spleen	Whole Blood
*MROH9*	0.0930	−0.1060	−0.2327	−0.0957	−11.815	−11.815
*CRP*	−0.0024	−0.1586	−0.2677	−0.0531	0.3596	−0.6280
*KIF26B*	0.3755	−0.3633	−0.4275	0.1002	−0.5859	−54.054
*NUBP1*	−0.0219	0.1659	0.0932	0.6114	0.7842	−0.4391
*SLC15A1*	−0.1079	−0.2001	−0.1035	−0.2850	−23.668	−68.529
*PKN1*	0.4406	0.6524	0.8633	0.6063	12.913	0.6124
*GBP3*	−11.977	13.194	19.294	0.7066	13.878	−11.340
*CCNG2*	−0.1532	0.6432	12.254	12.614	0.6959	−0.7247
*STAB1*	−0.0473	20.651	27.469	0.5974	40.382	0.9264
*GNAI2*	0.7099	0.7281	0.9906	0.2581	11.305	19.680
*PTGDR*	0.4130	0.9623	33.963	−0.0985	37.453	20.216
*PNN*	0.2193	12.600	18.740	0.9020	0.9617	−17.914
*VCAM1*	17.452	32.880	40.500	26.203	54.978	−52.102
Color scale	−3~−2	−2~−1	−1~+1	+1~+2	+2~+3	+3~

**Table 3:** Query genes from our exome analysis, interacting genes are from the Infevers database using the COXPRESdb.

Query Gene	Interact Gene	Data Source	Experiment Type	Pubmed
*GNAI2*	*UBA1*	HPRD_complex	in vivo	16263121
*PKN1*	*PSMB4*	IntAct(hsa)	validated two hybrid	32296183
*PKN1*	*PSMB4*	IntAct(hsa)	two hybrid prey pooling approach	32296183
*VCAM1*	*PSMA3*	IntAct(hsa)	cross-linking study	22623428
*VCAM1*	*TRAP1*	IntAct(hsa)	cross-linking study	22623428

**Table 4:** Analysis from the STRING database. Node 1 has genes from the exomes, node 2 has the genes from Infevers database. The threshold for the combined score was set to 0.4.

Node1	Node2	Co-Expression	Experimentally Determined Interaction	Database Annotated	Automated Text Mining	Combined Score
GNAI2	CDC42	0.249	0.352	0.900	0.167	0.954
PKN1	MEFV	0.057	0	0.800	0.517	0.901
CRP	IL10	0	0	0	0.838	0.838
CRP	VCAM1	0	0	0	0.802	0.802
VCAM1	IL10	0.053	0	0	0.708	0.712
SLC15A1	SLC29A3	0.066	0	0	0.679	0.688
CRP	IL1RN	0.062	0	0	0.681	0.687
PKN1	CDC42	0.062	0.300	0	0.504	0.646
CRP	TNFRSF1A	0	0	0	0.601	0.601
VCAM1	TNFRSF1A	0	0	0	0.595	0.595
GBP3	NLRP3	0.072	0	0	0.548	0.563
CRP	MEFV	0	0	0	0.556	0.556
CRP	NLRP3	0	0	0	0.556	0.556
CRP	IL36RN	0.062	0	0	0.541	0.551
GBP3	PSMB8	0.241	0.058	0	0.345	0.491
VCAM1	ADAM17	0.049	0	0	0.462	0.466
SLC15A1	NOD2	0	0	0	0.462	0.462
GNAI2	ALPK1	0	0	0	0.455	0.455
VCAM1	NLRP3	0.052	0	0	0.428	0.435
VCAM1	IL36RN	0	0	0	0.427	0.427
VCAM1	IL1RN	0.062	0	0	0.410	0.423
CRP	NOD2	0	0	0	0.407	0.407
VCAM1	CDC42	0.089	0	0	0.372	0.403

## Data Availability

Data sharing not applicable.
